# Predicting the Impact of Deleterious Single‐Nucleotide Polymorphisms in the p47ING1a Isoform of Human ING1 Gene

**DOI:** 10.1155/genr/2859448

**Published:** 2026-06-19

**Authors:** Md. Oliullah Rafi, Md. Takim Sarker, Mohammad Ashik Sheikh, Sowmitro Das, Sajal Kumar Halder, Md. Ashiqul Islam

**Affiliations:** ^1^ Department of Microbiology and Hygiene, Bangladesh Agricultural University, Mymensingh, 2202, Bangladesh, bau.edu.bd; ^2^ Department of Genetic Engineering and Biotechnology, Jashore University of Science and Technology, Jashore, 7408, Bangladesh, just.edu.bd; ^3^ UAB Heersink School of Medicine, Department of Biochemistry and Molecular Genetics, Birmingham, USA, uab.edu; ^4^ Department of Chemistry and Biochemistry, University of Windsor, Windsor, Canada, uwindsor.ca

**Keywords:** cancer genomics, H3K4me3, in silico analysis, ING1, nsSNPs, p47ING1a, PHD zinc finger, plant homeodomain, protein stability, tumor suppressor

## Abstract

**Purpose:**

The p47ING1a isoform of the *ING1* tumor suppressor regulates cellular senescence through Rb‐dependent pathways via its plant homeodomain (PHD) zinc‐finger, which recognizes the H3K4me3 histone mark. However, the mutational landscape of p47ING1a and the functional consequences of PHD‐domain nonsynonymous single‐nucleotide polymorphisms (nsSNPs) remain poorly characterized. This study aimed to identify and structurally evaluate the most deleterious nsSNPs in p47ING1a and clarify their potential role in disrupting ING1 tumor‐suppressor activity.

**Methods:**

A total of 347 missense nsSNPs were retrieved from the NCBI dbSNP database and screened using 12 sequence‐based computational tools. Variants consistently predicted as deleterious were further evaluated by I‐Mutant stability analysis and ConSurf evolutionary conservation profiling. Three‐dimensional structural modeling was performed using AlphaFold3, refined through GalaxyRefine, and validated by ERRAT, PROCHECK, and TM‐align. Mutation‐induced structural and binding effects were assessed using Missense3D, mCSM, and BeAtMuSiC. Post‐translational modification sites were predicted via NetPhos 3.1, GPS 3.0, BDM‐PUB, and NetOGlyc 4.0. Protein–protein interaction networks were constructed using STRING and Gene MANIA. Pan‐cancer expression was analyzed through UALCAN and the Human Protein Atlas.

**Results:**

Twelve computational tools converged on six high‐priority variants, namely, C358S, C374G, W378G, F379V, S382L, and R400P. All localized exclusively within the PHD zinc‐finger domain, residues 353–402. All six mutations were consistently predicted to destabilize the p47ING1a protein across multiple stability analyses.

**Conclusions:**

Six nsSNPs in the PHD domain of p47ING1a are predicted to disrupt protein stability, H3K4me3 binding, and Sin3A/HDAC complex interactions, thereby impairing ING1 tumor‐suppressor function. These findings provide a computational basis for prioritizing variants for experimental validation through site‐directed mutagenesis, chromatin‐binding assays, and structure‐guided therapeutic targeting of the PHD–H3K4me3 interface.

## 1. Introduction

The inhibitor of growth 1 (ING1) gene is a Type II tumor suppressor involved in regulating cell‐cycle progression, apoptosis, DNA repair, and cellular senescence through epigenetic mechanisms [1]. ING1 proteins function as chromatin regulators by interacting with histone acetyltransferase (HAT) and histone deacetylase (HDAC) complexes, and by recognizing histone H3 lysine 4 trimethylation (H3K4me3) via a conserved C‐terminal plant homeodomain (PHD) finger [2, 3], dysregulation or loss of ING1 expression has been reported in multiple human malignancies, underscoring its critical role in tumor suppression [4].

The ING1 gene, located at chromosome 13q34, encodes multiple alternatively spliced isoforms, among which p33ING1b (∼33 kDa) and p47ING1a (∼47 kDa) are the two major variants. These isoforms share conserved C‐terminal domains, including partial bromodomains (PBDs), lamin‐interacting domains (LIDs), nucleolar translocation signals (NTS), nuclear localization sequences (NLS), PHDs, and a polybasic region (PBR), but differ markedly in their N‐terminal regions. Notably, p47ING1a possesses an extended N‐terminal senescence‐associated domain (SAD) that is absent from the other isoforms and is required for its mitochondrial targeting. This domain enables p47ING1a to preferentially promote cellular senescence through Rb‐dependent but p53‐independent pathways, thereby inducing irreversible growth arrest under stress via coordinated transcriptional and mitochondrial mechanisms [4, 5].

Cellular senescence serves as a critical barrier against tumorigenesis by preventing the proliferation of damaged cells, and disruption of this process is a hallmark of cancer progression. Given the central role of p47ING1a in senescence regulation, mutations affecting this isoform may impair tumor‐suppressive mechanisms and facilitate oncogenic transformation [6]; however, compared with p33ING1b, the mutational landscape of p47ING1a remains largely unexplored [7].

Recent advances in computational analysis of nonsynonymous single‐nucleotide polymorphisms (nsSNPs) have enabled systematic evaluation of mutation‐induced structural and functional alterations [8]. In this study, we employed an integrated in silico approach using multiple predictive and structural bioinformatics tools to identify deleterious nsSNPs in p47ING1a and to assess their potential impact on protein stability, evolutionary conservation, and function, thereby providing insight into isoform‐specific tumor‐suppressor mechanisms.

## 2. Methods

**FIGURE 1:**
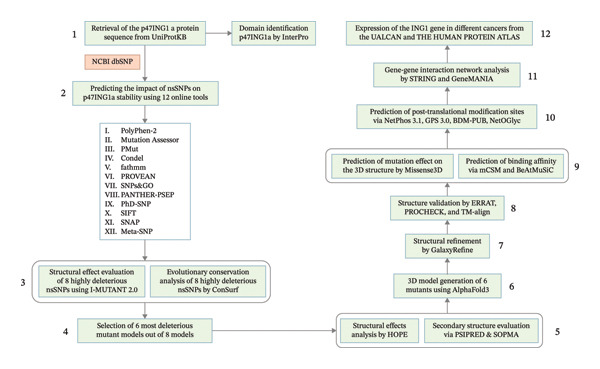
Schematic workflow of the study, showing nsSNP retrieval from NCBI dbSNP, identification of disease‐associated variants using 12 sequence‐based tools, and prediction of their structural stability effects on the p47ING1a protein.

The overall methodology of the study is summarized in Figure 1. Created by BioRender.

### 2.1. Collection of nsSNPs

SNP data for the human ING1 gene were retrieved from the NCBI dbSNP database [9]. Mutation IDs were collected by selecting variants under the function class category annotated as missense. The protein sequence was collected from the UniProt database [10] (UniProtKB ID Q9UK53).

### 2.2. Identification of Most Damaging nsSNPs

We used 12 computational tools to identify missense SNPs predicted to significantly affect ING1 protein structure and function.

*Sorting Intolerant From Tolerant (SIFT):* SIFT [11] is a web server that uses sequence homology to predict whether an amino acid substitution at a given position is likely to be damaging or tolerated. The substitution probability of < 0.05 at a particular amino acid position is considered intolerant and deleterious; a score of ≥ 0.05 is predicted as tolerated [12].

*PolyPhen-2:* PolyPhen‐2 [13] is a widely used computational tool that predicts the functional impact of SNPs on protein structure. This tool accepts the FASTA format of protein sequence as an input, and the prediction is performed based on the Bayesian classifier method [14]. The output score ranges from 0 (neutral) to 1 (deleterious), and functional significance was classified into probably damaging (0.85–1), possibly damaging (0.15–0.85), and benign (0.00–0.14) [15].

*PROVEAN:* PROVEAN [16] was utilized to evaluate the change in protein biological function caused by an amino acid substitution. It accepts the raw FASTA‐formatted sequence, identifies homologs using NCBI BLAST, and reports a numerical score. Scores greater than 2.5 are classified as neutral, and scores below 2.5 indicate deleterious effects.

*Mutation Assessor:* Mutation Assessor [17] can predict the functional effect of disease‐associated amino acids based on evolutionary conservation. To assess functional consequences of each mutation, the server uses the protein ID as input and classifies the impact of the amino acid substitution as high, medium, or low.

*PhD-SNP:* PhD‐SNP [18] is an SVM‐based classifier that categorizes neutral or disease‐associated nonsynonymous mutations from protein sequence. This algorithm uses a BLAST‐based sequence alignment against the UniRef90 database [19] to evaluate each variant. PhD‐SNP outputs a score between 0 and 1, where 0 indicates a disease‐associated nsSNP and 1 indicates a non–disease‐associated variant.

*SNPs&GO:* SNPs&GO [20] computes the deleterious effect of nsSNPs on the basis of functional and molecular information from the gene ontology database and gives a probability score. The probability score of ≥ 0.5 is predicted as a disease‐causing.

*SNAP:* SNAP [21] used the neural network classifier for predicting the functional impact of single amino acid substitution in the ING1 protein.

*PMut:* PMut [22] is a fast and accurate algorithm for predicting the pathogenicity of point mutations. It takes the protein FASTA sequence as input and returns a prediction score between 0 and 1, where values greater than 0.5 indicate that the nsSNP is likely to have a pathological effect on protein function.

*Condel:* Condel [23] classifies nonsynonymous SNPs as neutral or deleterious using a consensus score derived from Logre, MAPP, PolyPhen‐2, and SIFT. The Condel score ranges from 0 to 1, with higher values indicating more deleterious variants.

*fathmm:* A computational tool [24] that predicts pathogenic single amino acid substitutions in the human genome, using a quantitative score to distinguish benign from pathogenic point mutations. Scores greater than 0.5 indicate deleterious SNPs.

*PANTHER-PSEP:* PANTHER‐PSEP tool [25] predicts damaging or benign nsSNPs by utilizing evolutionary preservation of amino acid sequences. The PSEP score is classified into possibly damaging (200–450), probably damaging (450 <), and probably benign (< 200).

*Meta-SNP:* Meta‐SNP tool [26] uses a random forest‐based binary classifier for predicting disease‐causing SNPs. The server takes the protein sequence in FASTA format together with the specified amino acid substitutions as input.

### 2.3. Domain Identification of p47ING1a

**FIGURE 2:**
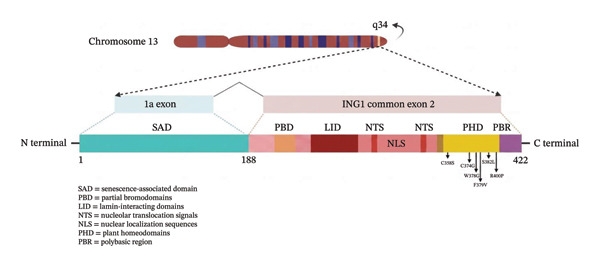
Genomic location and domain architecture of the p47ING1a isoform.

The location of nonsynonymous SNPs on the conserved domain of ING1 was identified by the InterPro web tool [27]. This tool can find domains and motifs of a protein and also recognize various functional characterizations of a protein, including protein functional sites, domains, and families [28]. We validated the domain architectures from Bertschmann et al. [5] (Figure 2). Domain architecture was created using the BioRender scientific illustration software [29].

### 2.4. Assessing nsSNP‐Induced Changes in Protein Stability

We assessed the impact of mutations on protein stability using the I‐Mutant 2.0 web server [30] (Table 1), a support vector machine–based tool trained on the ProTherm experimental thermodynamic database. We submitted 8 nsSNPs in FASTA format as input.

**TABLE 1:** Predicted effects of nsSNPs on p47ING1a protein stability using I‐Mutant.

Serial no.	SNP ID	Mutation	Stability	RI	DDG‐free energy change value (kcal/mol)
1	rs121909250	C358S	Decrease	8	−1.15
2	rs1028537467	S382L	Decrease	3	0.06
3	rs1357950159	G385R	Decrease	7	−0.44
4	rs1362770017	E226V	Increase	3	0.18
5	rs1438666150	R400P	Decrease	6	−0.63
6	rs1594457838	C374G	Decrease	5	−1.25
7	rs1594457853	W378G	Decrease	9	−2.17
8	rs1594457860	F379V	Decrease	7	−1.64

### 2.5. Conservation Analysis

**FIGURE 3:**
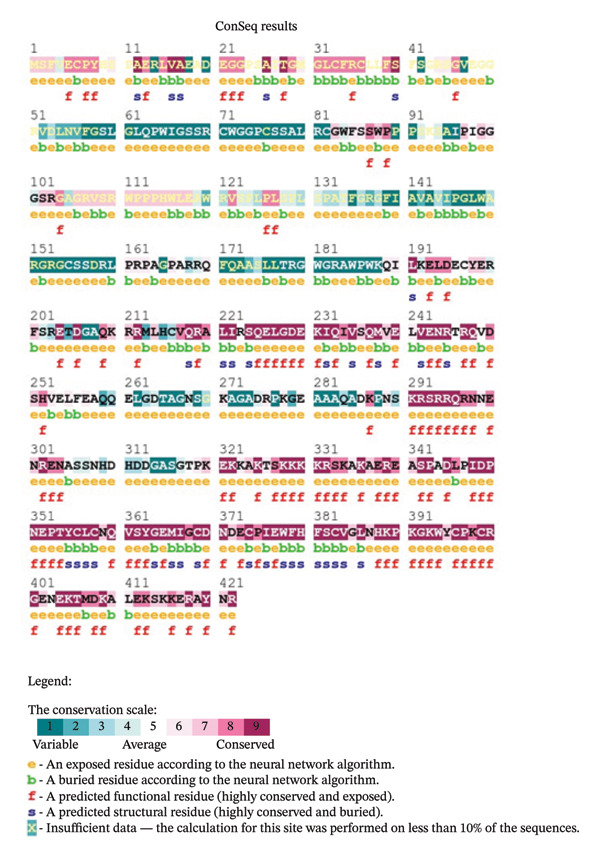
Evolutionary conservation profile of p47ING1a protein.

Evolutionary conservation of the amino acid in the protein sequences was identified using the ConSurf web server [31, 32] (Figure 3). ConSurf uses sequence homology to estimate the evolutionary conservation of amino acid residues. The server accepts the FASTA‐formatted protein sequence. The conservation score of amino acid residues is classified into 3 groups: a score of 1–4 indicates variable, 5–6 is intermediate, and 7–9 is considered conserved. nsSNPs predicted by I‐Mutant 2.0 to destabilize the protein and those with high ConSurf conservation scores were selected for further analysis.

### 2.6. Structural Effect Analysis of nsSNP

The HOPE web server was used to evaluate the structural consequences of ING1 nsSNPs. HOPE analyzes point mutations using available tertiary structure information, integrating annotations from UniProt and the distributed annotation system (DAS) to predict their impact on the protein [33].

### 2.7. Structural Evaluation of Native and Mutant Proteins

The secondary structural evaluation of the mutant and wild‐type ING1 was performed using PSIPRED [34] and SOPMA [35] tools (Supporting Table 2). PSIPRED uses Position‐Specific Iterated‐BLAST (PSI‐BLAST) to predict the secondary structure of a protein. The SOPMA server predicts the secondary structure of a protein by assigning residues to four states: helix, sheet, turn, or coil. The 3D structure of mutated ING1 with high‐risk nsSNPs and native proteins was modeled using AlphaFold 3 [36]. The best structure obtained from AlphaFold 3 was further refined using the GalaxyRefine web server [37]. GalaxyRefine rebuilds and repacks side chains, then relaxes the model by molecular dynamics, yielding consistent improvements in both global and local structure quality. Refined 3D models from GalaxyRefine were validated using quality factor scores from ERRAT in the SAVES v6.1 online server [38], Ramachandran plot analysis via PROCHECK [39], and TM‐align tool [40] for the comparison of native versus mutant structures (TM‐score 0–1, with higher TM‐scores and lower RMSD values indicating closer structural similarity).

### 2.8. Prediction of Mutation‐Induced Structural and Binding Effects

To validate our predicted mutants, we also included previously characterized ING1 PHD‐finger variants from earlier studies, namely, Y355A, N359S, V361I, G364V, and W378A. Because most prior functional work focused on the p33ING1b isoform, these residues correspond to Y212A, N216S, V218I, G221V, and W235A in p33ING1b amino acid numbering [41]. We used Missense3D [42] to assess structural alterations induced by missense variants in the p47ING1a tertiary structure. Furthermore, we applied mCSM [43], which predicts mutation effects using graph‐based signatures, together with BeAtMuSiC [44], a coarse‐grained predictor of mutation‐induced changes in binding free energy.

### 2.9. Prediction of Post‐Translational Modifications (PTMs) Sites

PTMs such as phosphorylation, glycosylation, and ubiquitination play key roles in regulating ING1 stability, localization, and tumor‐suppressor function [45–47]. Phosphorylation sites at threonine (T), serine (S), and tyrosine (Y) residual position of p47ING1a protein were predicted using NetPhos 3.1 [48] and GPS 3.0 [49]. NetPhos 3.1 employs an ensemble of neural networks to predict phosphorylation sites at specific residues using a default score cutoff of 0.5. GPS 3.0 applies a group‐based phosphorylation scoring approach to predict kinase‐specific phosphorylation sites directly from protein primary sequences, covering 71 distinct protein kinase groups. Amino acid positions with a prediction score greater than 0.5 were considered phosphorylated. Ubiquitylation sites in the ING1 protein were predicted from the BDM‐PUB server [50]. Glycosylation, another key PTM, was evaluated using the NetOGlyc 4.0 server to predict O‐linked glycosylation sites [51]. Residues with scores above 0.5 were considered glycosylated.

### 2.10. Gene–Gene Interaction Network

The STRING v.11.0 [52] and GENE MANIA [53] servers were used to study the interaction and association of the ING1 gene with other genes. String predicts and analyzes the interaction of genes based on homology, text mining, database, experiments, coexpression, concurrence, gene fusion, and neighborhood. STRING confidence scores ranged from 0 to 1, with higher values indicating stronger predicted interactions. Gene MANIA predicts functional interaction and association networks on the basis of protein domain similarity index, physical interaction, genetic interaction, pathway, co‐localization, and coexpression.

### 2.11. Expression Pattern of ING1 in Different Cancer Types

The expression of the *ING1* gene across cancers was assessed from the TCGA database through the UALCAN [54] online tool. Protein expression in the relevant cancers was also assessed using the Human Protein Atlas, which integrates tissue microarray‐based immunohistochemistry with transcriptomic profiling across human tissues [55, 56].

## 3. Results

### 3.1. SNP Annotation

**FIGURE 4:**
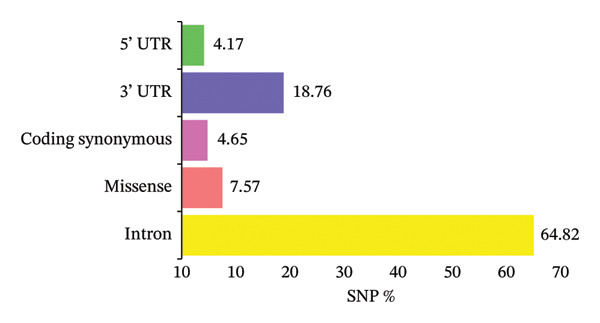
Distribution of the nsSNPs in 3′ UTR, 5′ UTR, coding synonymous, nonsynonymous missense, and intronic region of the ING1 gene.

The National Centre for Biotechnology Information, NCBI dbSNP database, was used to retrieve the polymorphism information of the human ING1 gene, which contained a total of 4187 SNPs for the ING1 protein. Out of 4187 SNPs, 862 are in the 3′ UTR region, 192 are in the 5′ UTR region, 214 are in coding synonymous, 348 are in nonsynonymous missense, and 2978 are in the intron region (Figure 4). In this study, only nonsynonymous missense SNPs of the ING1 gene were subjected to further analysis, as they alter the encoded amino acid.

### 3.2. Determination of Most Damaging SNPs

**FIGURE 5:**
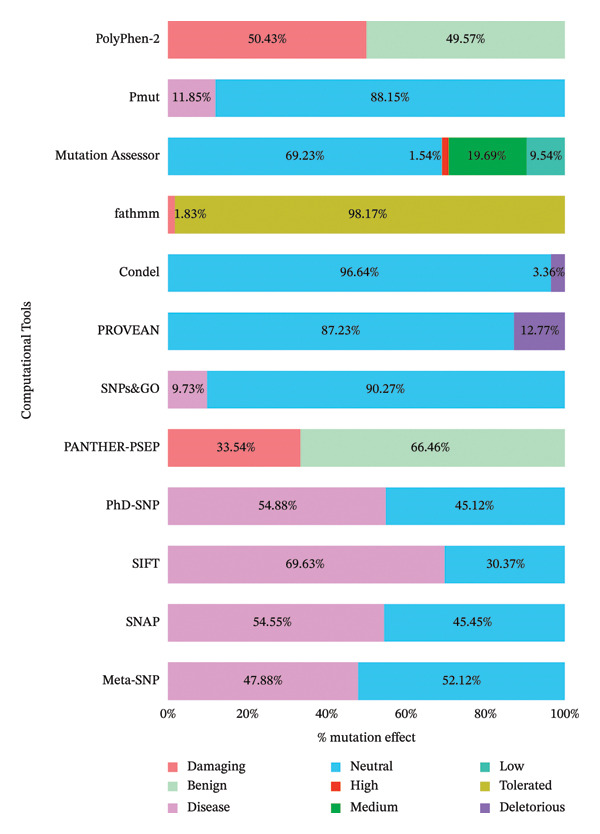
Predicted effects of 347 nonsynonymous SNPs in the ING1 gene using 12 computational tools.

In order to identify the most damaging SNPs that significantly alter the function and structure of ING1 protein, we utilized 12 different computational tools (PolyPhen‐2, PMut, Mutation Assessor, fathmm, Condel, PROVEAN, SNPs&GO, PANTHER‐PSEP, PhD‐SNP, SIFT, SNAP, Meta‐SNP) (Supporting Material 1). Among 347 missense variants, PolyPhen‐2 flagged 175 nsSNPs as probably damaging, and multiple additional predictors (PMut, MutationAssessor, FATHMM, PROVEAN, Condel, SNPs&GO, Panther‐PSEP, PhD‐SNP, SIFT, SNAP, and Meta‐SNP) consistently identified overlapping sets of 5–188 nsSNPs with predicted deleterious or disease‐causing effects on ING1 (Figure 5). Integrating all prediction tools, only 8 of the 347 nsSNPs were consistently classified as highly deleterious (Table 2), and these variants were selected for downstream analyses.

**TABLE 2:** Most damaging nonsynonymous SNPs (nsSNPs) in the ING1gene were predicted as deleterious by all 12 computational tools.

Rs ID	Mutation	PolyPhen‐2	PMut	Mutation assessor	Fathmm	Condel	PROVEAN	SNPs&GO	PANTHER‐PSEP	PhD‐SNP	SIFT	SNAP	Meta‐SNP
rs121909250	C358S	Probably Damaging	Disease	High	Damaging	Disease	Deleterious	Disease	Probably Damaging	Disease	Disease	Disease	Disease
rs1028537467	S382L	Probably Damaging	Disease	Medium	Tolerated	Disease	Deleterious	Disease	Probably Damaging	Disease	Disease	Disease	Disease
rs1357950159	G385R	Probably Damaging	Disease	Medium	Tolerated	Disease	Deleterious	Disease	Probably Damaging	Disease	Disease	Disease	Disease
rs1362770017	E226V	Probably Damaging	Disease	Medium	Tolerated	Disease	Deleterious	Disease	Probably Damaging	Disease	Disease	Disease	Disease
rs1438666150	R400P	Probably Damaging	Disease	High	Damaging	Disease	Deleterious	Disease	Probably Damaging	Disease	Disease	Disease	Disease
rs1594457838	C374G	Probably Damaging	Disease	High	Damaging	Disease	Deleterious	Disease	Probably Damaging	Disease	Disease	Disease	Disease
rs1594457853	W378G	Probably Damaging	Disease	High	Tolerated	Disease	Deleterious	Disease	Probably Damaging	Disease	Disease	Disease	Disease
rs1594457860	F379V	Probably Damaging	Disease	High	Tolerated	Disease	Deleterious	Disease	Probably Damaging	Disease	Disease	Disease	Disease

### 3.3. Domain Identification in ING1

The p47ING1a gene is located in the subtelomeric region of chromosome 13q34 and is 422 amino acids long (Figure 2). It encodes shared conserved C‐terminal domains, including a PBD, LID, NTS, nuclear localization signal (NLS), PHD, and a PBR, but differ substantially in their N‐terminal regions.

Notably, p47ING1a contains an extended, largely unstructured N‐terminal SAD (residues 1–188 and encoded by the exon 1a) that includes an amphipathic segment, a protease cleavage site, and TOM20 recognition motifs typical of mitochondria‐targeted proteins. The PHD zinc‐finger domain, which binds the histone H3K4me3 mark associated with active transcription, is the most highly conserved region among ING family proteins [5]. All 6 of the final predicted mutations are located within the PHD domain (353–402 aa [57]).

### 3.4. Predicting Impacts of nsSNPs on Protein Stability

The effect of each variant on p47ING1a protein stability was evaluated using reliability index (RI) and ΔΔ*G* values from I‐Mutant. According to these predictions (Table 1), only the E226V substitution is associated with increased stability, whereas all other analyzed mutations are predicted to destabilize the protein.

### 3.5. Conservation Analysis

The ConSurf server identifies putative structural and functional amino acids and recognizes their evolutionary conservation profile based on the Bayesian method. ConSurf analysis identified seven residues with the highest conservation score (Table 3). C358, S382, C374, W378, and F379 are predicted as buried and structural, whereas R400 and E226 are exposed and functional (Figure 3). In contrast, G385 is moderately conserved and buried. Based on the high conservation and predicted destabilizing effects from ConSurf and I‐MUTANT 2.0, six highly deleterious nsSNPs (C358S, C374G, W378G, F379V, S382L, R400P) were selected for further analysis.

**TABLE 3:** Conservation profile of eight high‐risk nsSNPs in p47ING1a.

Serial no.	SNP ID	Mutation	Conservation score	Prediction
1	rs121909250	C358S	9	Highly conserved and buried (s)
2	rs1028537467	S382L	9	Highly conserved and buried (s)
3	rs1357950159	G385R	5	Average conserved Buried
4	rs1362770017	E226V	9	Highly conserved and exposed (f)
5	rs1438666150	R400P	9	Highly conserved and exposed (f)
6	rs1594457838	C374G	9	Highly conserved and buried (s)
7	rs1594457853	W378G	9	Highly conserved and buried (s)
8	rs1594457860	F379V	9	Highly conserved and buried (s)

### 3.6. Structural Effect Analysis of Nonsynonymous SNP on Human ING1 Protein

The mutations C374G, W378G, F379V, and R400P replace the wild‐type residues with smaller amino acids, whereas S382L introduces a larger side chain than in the native residue (Supporting Table 1). Moreover, C358S, C374G, and W378G reduce side‐chain hydrophobicity relative to the wild type, whereas S382L and R400P increase it, and the R400P substitution also changes the residue from positively charged to neutral.

### 3.7. Structural Evaluation of Native and Mutant Protein

**FIGURE 6:**
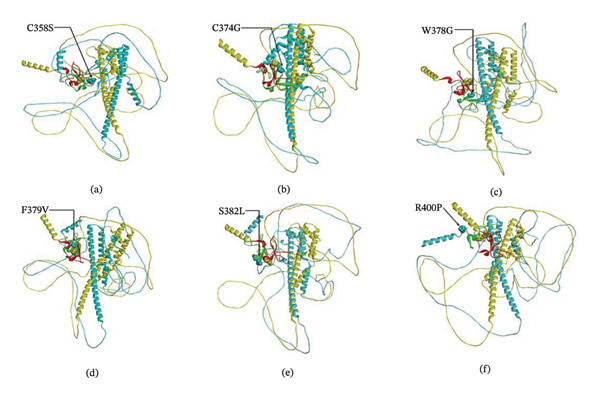
Superimposed 3D models of the six p47ING1a mutants. The wild‐type backbone is shown in yellow and mutant backbones in cyan; PHD domains are highlighted in red (wild type) and green (mutants), with mutation sites marked as sphere representations.

PSIPRED predicted the wild‐type protein as predominantly coil (59.47%), with 33.88% alpha‐helix and 6.63% beta‐sheet (Supporting Table 2). Notably, in the C374G mutant, coil content increased to 62.79%, while alpha‐helix content decreased to 30.56% compared with the wild type. SOPMA predicted the native ING1 protein to comprise 31.75% alpha‐helix, 11.61% extended strand, 4.27% beta turn, and 52.37% random coil, with all mutant variants showing noticeable shifts in these secondary structure proportions. To assess whether the most damaging nsSNPs alter ING1 structure, we modeled the 3D structures of the wild‐type protein and six mutants (C358S, C374G, W378G, F379V, S382L, R400P) using AlphaFold3 (Figure 6). Further refinement of the models was done using the Galaxy Refine server based on the structural properties listed in Supporting Table 3. Structural validation reports from ERRAT showed reliable quality scores ranging from 90 to ∼100 [58]. The Ramachandran plot predicted more than 93% residues in the most favored region (Table 4). In addition, structural comparisons between native and mutant models were studied based on RMSD and TM‐score utilizing the TM‐align tool. In Supporting Table 4, F379V shows an RMSD of 6.08 and a TM‐score of 0.33046, whereas C358S has an RMSD of 4.12 and a TM‐score of 0.32110, indicating the largest and smallest structural deviations among the mutants, respectively, based on TM‐align; in both cases, the relatively low aligned lengths likely contribute to the higher apparent variability of these models.

**TABLE 4:** Structural validity analysis in the SAVES v6.1 web server.

Model	ERRAT	PROCHECK—Ramachandran plot
	Quality factor	Most favored regions (%)
WT	99.537	93.7
C358S	90.1478	94.5
C374G	91.8182	96.3
W378G	93.9698	93.7
F379V	99.5261	94.8
S382L	95.7746	94.0
R400P	93.8889	94.2

*Note:* ERRAT and Ramachandran plot assessment of the finalized native and mutant models of p47ING1a protein.

### 3.8. Prediction of Mutation‐Induced Structural and Binding Effects

Missense3D predicted three of the eleven analyzed variants (G364V, F379V, R400P; five literature‐derived and six newly identified) as structurally damaging in p47ING1a (Supporting Table 5). Using the crystal structure of the ING1 PHD finger bound to H3K4me3 (PDB ID: 2QIC) [59] as input, BeAtMuSiC indicated significantly reduced binding affinity for four interface mutants: Y355A, G364V, W378G, and W378A (Supporting Table 6). Consistently, mCSM predicted all mutants selected in our pipeline to destabilize the protein (Supporting Table 7).

### 3.9. PTM Site Prediction

NetPhos 3.1 predicted 38 phosphorylation sites in p47ING1a: 31 serine, 4 threonine, and 3 tyrosine residues. GPS 5.0 displayed 14 amino acid positions as phosphorylated (Ser: 12 and Thr: 2) (Supporting Table 8). Only ten serine residue positions were found to match between the two servers. BDM‐PUB predicted 24 lysine residues that can undergo ubiquitylation (Supporting Table 9). Possible glycosylation sites were identified using the NetOGlyc 4.0 server, as represented in Supporting Table 10. The predictions indicate that the mutant proteins are more extensively glycosylated than the wild type: Five of the six mutants gain a glycosylation site at Position 265 (all except W378G), whereas the wild‐type protein remains unglycosylated.

### 3.10. Gene–Gene Interaction Network of ING1

**FIGURE 7:**
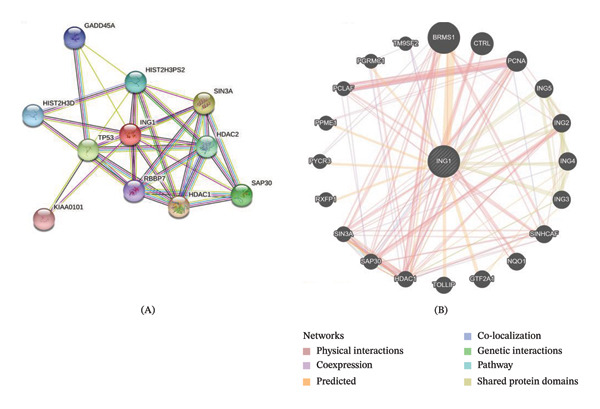
The interaction pattern of the ING1 gene with other genes. The gene network was created by (A) STRING and (B) Gene MANIA database.

STRING identified 10 putative interaction partners for ING1, with HDAC1 showing the highest combined confidence score (0.998) based on text mining, coexpression, and experimental evidence. In addition, HDAC1, SIN3A, HDAC2, HIST2H3PS2, HIST2H3D, and RBBP7 are coexpressed with ING1(Supporting Table 11). HDAC1, together with SIN3A, represents the strongest predicted interactors in the network (Figure 7A). Consistent with previous reports that ING1 is a stable component of mSIN3A/HDAC1‐containing chromatin–remodeling complexes, these interactions support a role for ING1 in histone deacetylation‐mediated transcriptional regulation [60]. GeneMANIA analysis (Supporting Table 12) indicated that four additional ING family members share protein domains with ING1, nine genes show physical interactions, and three genes are coexpressed with ING1.

### 3.11. Expression Pattern of ING1 in Different Cancer Types

**FIGURE 8:**
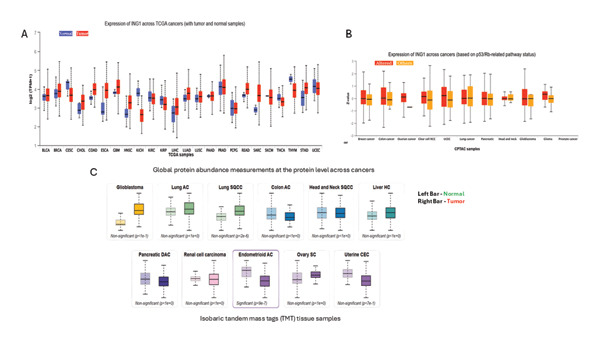
ING1 expression across human cancers. (A) mRNA expression of ING1 in tumor versus normal tissues across TCGA cohorts from UALCAN. (B) ING1 mRNA expression stratified by p53/Rb pathway status. (C) ING1 protein abundance in CPTAC tumor and normal tissues from the Human Protein Atlas (TMT‐based mass spectrometry).

ING1 expression and clinical outcome were examined across The Cancer Genome Atlas (TCGA) cancers using UALCAN and validated at the protein level with Clinical Proteomic Tumor Analysis Consortium (CPTAC) and Human Protein Atlas data (Figure 8). Pan‐cancer analysis showed that ING1 mRNA is broadly expressed but variably dysregulated among tumor types relative to matched normal tissues. Stratification by p53/Rb‐related pathway status indicated higher ING1 levels in tumors with pathway alterations. Consistently, proteomic profiles derived from CPTAC TMT‐based mass spectrometry in the Human Protein Atlas revealed cancer‐type‐specific differences in ING1 abundance, with significant upregulation in endometrioid adenocarcinoma and more modest, nonsignificant changes in several other cancers. Together, these transcriptomic and proteomic data indicate a context‐dependent role of ING1 in cancer, characterized by heterogeneous dysregulation rather than uniform loss across tumor types.

## 4. Discussion

This study employed a multitiered computational pipeline to systematically identify, prioritize, and structurally characterize the most deleterious nsSNPs in the ING1 gene, with a specific focus on the p47ING1a isoform. Beginning from 347 missense variants retrieved from the NCBI dbSNP database, sequential filtering through 12 sequence and structure‐based tools converged on six high‐risk variants C358S, C374G, W378G, F379V, S382L, and R400P, all mapping to the PHD domain (residues 353–402). Secondary structure analysis, protein stability predictions, evolutionary conservation profiling, PTM mapping, binding affinity assessments, and pan‐cancer expression analyses collectively reinforce the pathogenic potential of these variants and provide isoform‐specific insight not previously available in the literature.

Previous mutational and functional studies of ING1 have largely centered on the p33ING1b isoform, whereas the nsSNP profile of p47ING1a remains poorly characterized despite its unique SAD, which mediates mitochondrial targeting and p53‐independent activation of the Rb checkpoint pathway. Mutations within p47ING1a may therefore impair cellular senescence even when p33ING1b function is intact [6]. This study addresses a meaningful gap in the understanding of ING1 isoform‐specific tumor suppressor biology.

All six prioritized variants localize to the highly conserved PHD zinc‐finger domain, which is essential for H3K4me3 recognition and ING1‐mediated DNA repair and apoptosis. Peña et al. (2008) showed that substitutions at Y212 and W235 (Y355 and W378 in p47ING1a) abolish H3K4me3 binding, while cancer‐associated N216S, V218I, and G221V mutations (N359S, V361I, and G364V in p47ING1a) impair nucleotide excision repair and apoptosis [41]. These reported variants, together with the six newly predicted mutations, were evaluated using Missense3D, BeAtMuSiC, and mCSM, which consistently identified several variants as structurally damaging or destabilizing. Notably, W378G is analogous to the functionally null W235A mutation and is therefore predicted to severely disrupt H3K4me3 binding. I‐Mutant predicted all six selected variants to be destabilizing, while cysteine substitutions (C358S, C374G) are particularly damaging because these residues coordinate zinc ions required for proper PHD folding [61]. ConSurf assigned all six residues the highest conservation score (9), confirming their functional importance across the ING family. Buried substitutions such as C374G, W378G, and F379V are predicted to destabilize the domain core, whereas the exposed R400P mutation likely disrupts surface interactions and backbone flexibility. Secondary structure analyses by PSIPRED and SOPMA, and TM‐align comparisons showed measurable conformational changes, with C374G displaying the strongest structural deviation. PTM prediction identified extensive phosphorylation potential and suggested that five mutants gain an aberrant O‐linked glycosylation site at residue 265, potentially altering turnover or protein interactions. STRING network analysis identified HDAC1, SIN3A, HDAC2, HIST2H3D, and RBBP7 as major ING1‐associated partners, consistent with its role in Sin3A/HDAC chromatin–remodeling complexes [62]. Because ING1 recruits HDAC activity to H3K4me3‐marked chromatin through its PHD domain, mutations disrupting this interaction may impair transcriptional repression, cell‐cycle regulation, and apoptosis. Pan‐cancer transcriptomic analysis via UALCAN revealed variable ING1 expression across TCGA tumor types relative to matched normal tissues, with elevated levels in p53/Rb pathway‐altered cancers consistent with a compensatory stress–response model previously described in oral squamous cell carcinoma [63]. Notably, CPTAC proteomic data from the Human Protein Atlas identified significant ING1 protein upregulation specifically in endometrioid adenocarcinoma (*p* < 9e^−7^), suggesting that cancer‐type‐specific regulatory mechanisms warrant further investigation in endometrial cancer biology. **Strengths:** This study is strengthened by the integration of 12 independent prediction tools, which improves confidence in identifying truly deleterious variants by reducing false‐positive calls. Unlike previous reports focused on p33ING1b, the present work provides an isoform‐specific analysis of p47ING1a, a biologically distinct and underexplored variant involved in senescence regulation. Additional robustness was achieved through cross‐validation with previously characterized PHD‐domain mutations and the combined use of advanced structural tools, including Missense3D, BeAtMuSiC, mCSM, AlphaFold3, and GalaxyRefine. Incorporation of TCGA and CPTAC pan‐cancer datasets further enhanced the clinical relevance of the findings. **Limitations:** The study is based entirely on computational predictions and therefore requires *in vitro* and *in vivo* validation to confirm the functional effects of prioritized variants. Population‐level allele frequencies and clinical prevalence of the selected mutations were not comprehensively assessed, limiting epidemiological interpretation. Structural models represent static conformations and do not capture dynamic protein behavior or mutation‐driven interaction changes under physiological conditions. In addition, predicted PTMs and pan‐cancer expression associations remain correlative and require experimental confirmation. **Future investigations** should validate the prioritized p47ING1a variants through site‐directed mutagenesis, chromatin‐binding experiments, and cellular models to assess their impact on senescence induction, Rb pathway activity, and cell‐cycle control. Molecular dynamics simulations may further clarify how these mutations alter PHD‐domain stability and H3K4me3 binding beyond static structural predictions. In addition, clinical sequencing studies and structure‐guided drug screening could assess the relevance of ING1 variants in cancer and identify compounds targeting the PHD–H3K4me3 interface.

## 5. Conclusion

This study applied a comprehensive in silico strategy to identify functionally significant nsSNPs in the p47ING1a isoform of the ING1 tumor‐suppressor gene. Among 347 missense variants, six high‐priority mutations (C358S, C374G, W378G, F379V, S382L, and R400P) were consistently predicted as deleterious and were all localized within the highly conserved PHD zinc‐finger domain, a critical region required for H3K4me3 recognition and ING1‐mediated DNA repair, apoptosis, and senescence. Structural and stability analyses demonstrated that these substitutions are likely to disrupt domain integrity and impair protein function. Collectively, these findings provide the first isoform‐specific mutational landscape of p47ING1a and highlight the biological importance of its PHD domain in tumor suppressor activity. This work establishes a foundation for future experimental validation and suggests that targeting the PHD–H3K4me3 interface may represent a promising therapeutic avenue in ING1‐associated malignancies.

## Author Contributions

Conceptualization: Md. Oliullah Rafi, Md. Takim Sarker, Mohammad Ashik Sheikh, and Sowmitro Das.

Formal analysis: Md. Oliullah Rafi, Md. Takim Sarker, Sajal Kumar Halder, Mohammad Ashik Sheikh, Sowmitro Das, Investigation: Md. Oliullah Rafi, Md. Takim Sarker, Sajal Kumar Halder, Mohammad Ashik Sheikh, and Sowmitro Das.

Methodology: Md. Oliullah Rafi, Md. Takim Sarker, Mohammad Ashik Sheikh, and Sowmitro Das.

Software: Md. Oliullah Rafi, Md. Takim Sarker, Sajal Kumar Halder, Mohammad Ashik Sheikh, and Sowmitro Das.

Supervision: Md. Takim Sarker.

Writing–original draft: Md. Oliullah Rafi, Md. Takim Sarker, and Sajal Kumar Halder.

Writing–review and editing: Md. Oliullah Rafi, Md. Takim Sarker, Sajal Kumar Halder, Mohammad Ashik Sheikh, and Sowmitro Das.

## Funding

No funding was received for this manuscript.

## Conflicts of Interest

The authors declare no conflicts of interest.

## Supporting Information

Additional supporting information can be found online in the Supporting Information section.

## Supporting information


**Supporting Information** Supporting Table 1: Structural effect of 6 nsSNPs over p47ING1a protein using Project Hope. Supporting Table 2: Secondary structural properties of wild‐type and 6 mutant proteins. The secondary structure was evaluated by SOPMA and PSIPRED tools. Supporting Table 3: Structural quality assessment of the refined model generated by the GalaxyRefine tool, including GDT‐HA, RMSD, MolProbity, clash score, poor rotamers, and Ramachandran favored region. Supporting Table 4: RMSD value and TM‐score of 6 most damaging nsSNPs of ING1 protein using TM‐align. Supporting Table 5: Assessment of 11 missense mutations on the protein’s tertiary structure. Supporting Table 6: Predicted binding affinity changes upon mutation in the ING1 (PHD–H3K4me3) complex. Supporting Table 7: Impact of mutation‐induced changes on the protein–protein binding affinity. Supporting Table 8: Prediction of phosphorylation sites by NetPhos 3.1 and GPS 3.0. Supporting Table 9: Prediction of ubiquitylation site by BDM–PUB server. Supporting Table 10: Prediction of glycosylation sites by NetOGlyc 4.0 server. Supporting Table 11: Functional protein partners of ING1 predicted by STRING. Supporting Table 12: Interaction of ING1 with other genes and their network group. Supporting Material 1: Prediction result of 347 ING1 nsSNPs by 12 computational tools (PolyPhen‐2, Pmut, Mutation Assessor, fathmm, Condel, PROVEAN, SNPs&GO, Panther‐PSEP, PhD‐SNP, SIFT, SNAP, Meta‐SNP).

## Data Availability

The datasets supporting the conclusions of this study are included within the article (and its additional files).
