# Supplementary material for: Predicting the Impact of Deleterious Single‐Nucleotide Polymorphisms in the p47ING1a Isoform of Human ING1 Gene
Source: Genet Res (Camb). 2026 Jun 19;2026:2859448. doi: 10.1155/genr/2859448 (PMC13282558; doi:10.1155/genr/2859448)
Supplement: Supplementary file 1 — Supporting Information Supporting Table 1: Structural effect of 6 nsSNPs over p47ING1a protein using Project Hope. Supporting Table 2: Secondary structural properties of wild‐type and 6 mutant proteins. The secondary structure was evaluated by SOPMA and PSIPRED tools. Supporting Table 3: Structural quality assessment of the refined model generated by the GalaxyRefine tool, including GDT‐HA, RMSD, MolProbity, clash score, poor rotamers, and Ramachandran favored region. Supporting Table 4: RMSD value and TM‐score of 6 most damaging nsSNPs of ING1 protein using TM‐align. Supporting Table 5: Assessment of 11 missense mutations on the protein’s tertiary structure. Supporting Table 6: Predicted binding affinity changes upon mutation in the ING1 (PHD–H3K4me3) complex. Supporting Table 7: Impact of mutation‐induced changes on the protein–protein binding affinity. Supporting Table 8: Prediction of phosphorylation sites by NetPhos 3.1 and GPS 3.0. Supporting Table 9: Prediction of ubiquitylation site by BDM–PUB server. Supporting Table 10: Prediction of glycosylation sites by NetOGlyc 4.0 server. Supporting Table 11: Functional protein partners of ING1 predicted by STRING. Supporting Table 12: Interaction of ING1 with other genes and their network group. Supporting Material 1: Prediction result of 347 ING1 nsSNPs by 12 computational tools (PolyPhen‐2, Pmut, Mutation Assessor, fathmm, Condel, PROVEAN, SNPs&GO, Panther‐PSEP, PhD‐SNP, SIFT, SNAP, Meta‐SNP). [file GENR-2026-2859448-s001.zip › Supplementary-Materials.docx]

ARTICLE

**Predicting the impact of deleterious single-nucleotide polymorphisms in the p47ING1a isoform of human ING1 gene**

Md. Oliullah Rafi ^a,†^, Md. Takim Sarker ^b,†^, Mohammad Ashik Sheikh ^b,†^ , Sowmitro Das ^b, †^, Sajal Kumar Halder ^c^, Md. Ashiqul Islam^d^

*^a^Department of Microbiology and Hygiene, Bangladesh Agricultural University, Mymensingh - 2202, Bangladesh*

*^b^Department of Genetic Engineering and Biotechnology, Jashore University of Science and Technology, Jashore, 7408, Bangladesh*

*^c^UAB Heersink School of Medicine, Department of Biochemistry and Molecular Genetics, Birmingham,* *United States*

*^d^Department of Chemistry and Biochemistry, University of Windsor, Canada*

*^†^ These authors contributed equally to this work.*

***Corresponding Author**

**Md. Oliullah Rafi**

Department of Microbiology and Hygiene, Bangladesh Agricultural University, Mymensingh - 2202, Bangladesh

E-mail: [rafi.btech.bd@gmail.com](mailto:rafi.btech.bd@gmail.com)

**Supplementary Tables**

**Supplementary Table 1**: Structural effect of 6 nsSNPs over p47ING1a protein using Project Hope.

| **Residue** | **Structure** | **Properties** |
| --- | --- | --- |
| **C358S** | 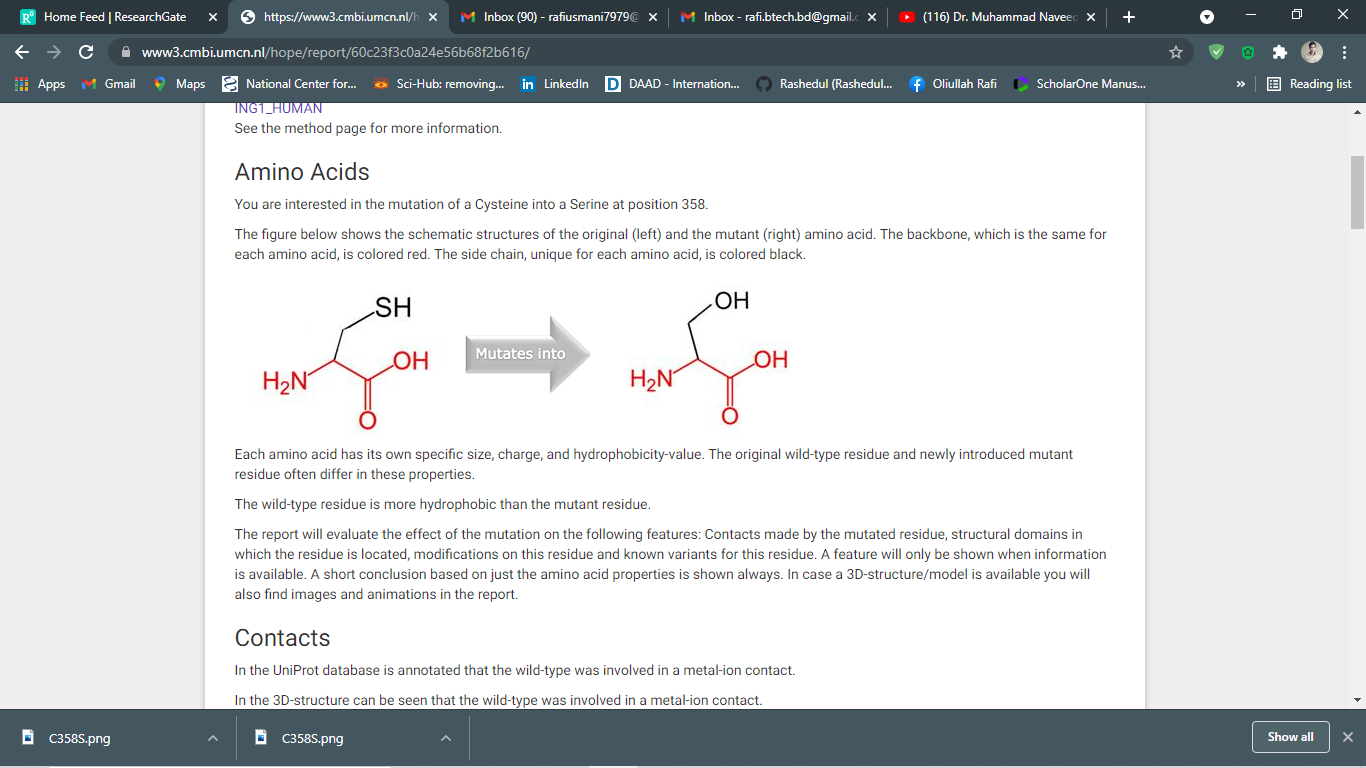 | -Each amino acid has its own specific size, charge, and hydrophobicity-value.  -The original wild-type residue and newly introduced mutant residue often differ in these properties.  -The wild-type residue is more hydrophobic than the mutant residue. |
| **C374G** | 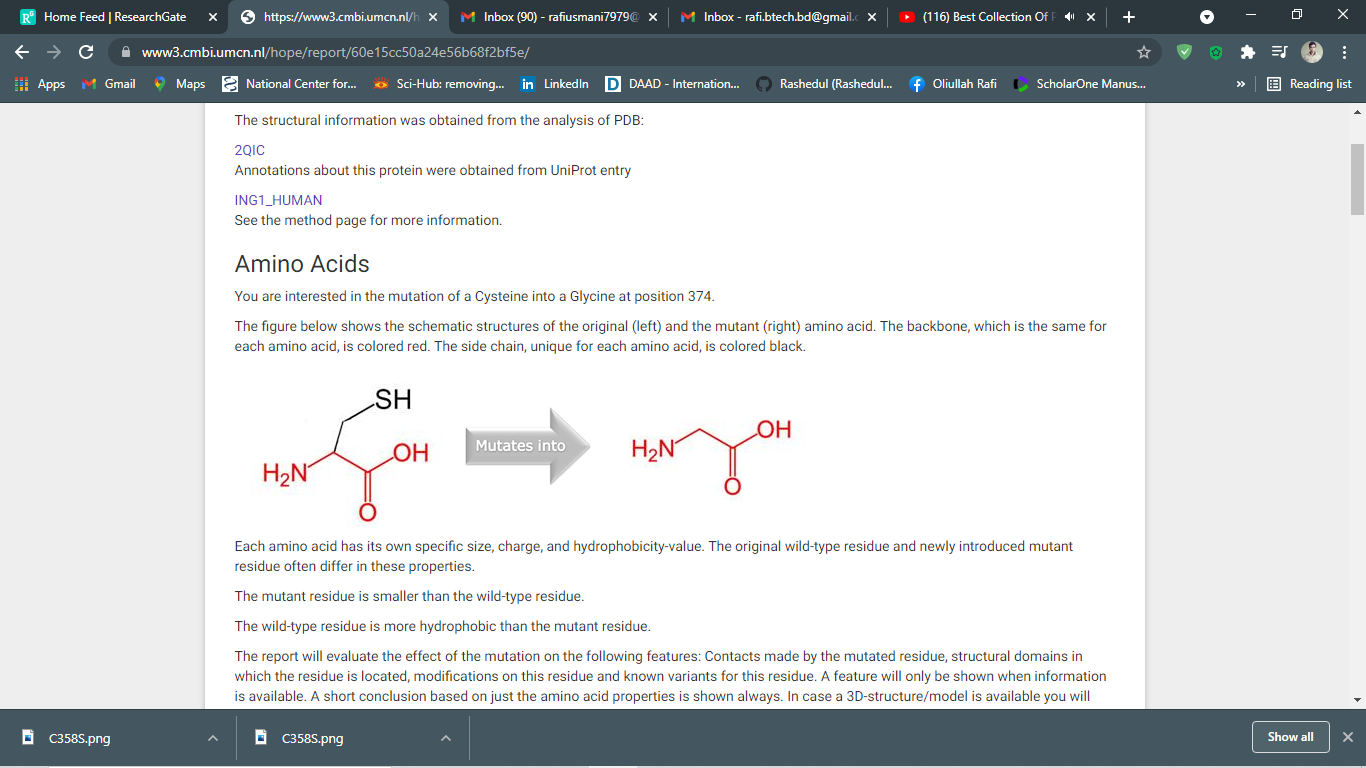 | -Each amino acid has its own specific size, charge, and hydrophobicity-value.  -The original wild-type residue and newly introduced mutant residue often differ in these properties.  -The mutant residue is smaller than the wild-type residue.  -The wild-type residue is more hydrophobic than the mutant residue. |
| **W378G** | 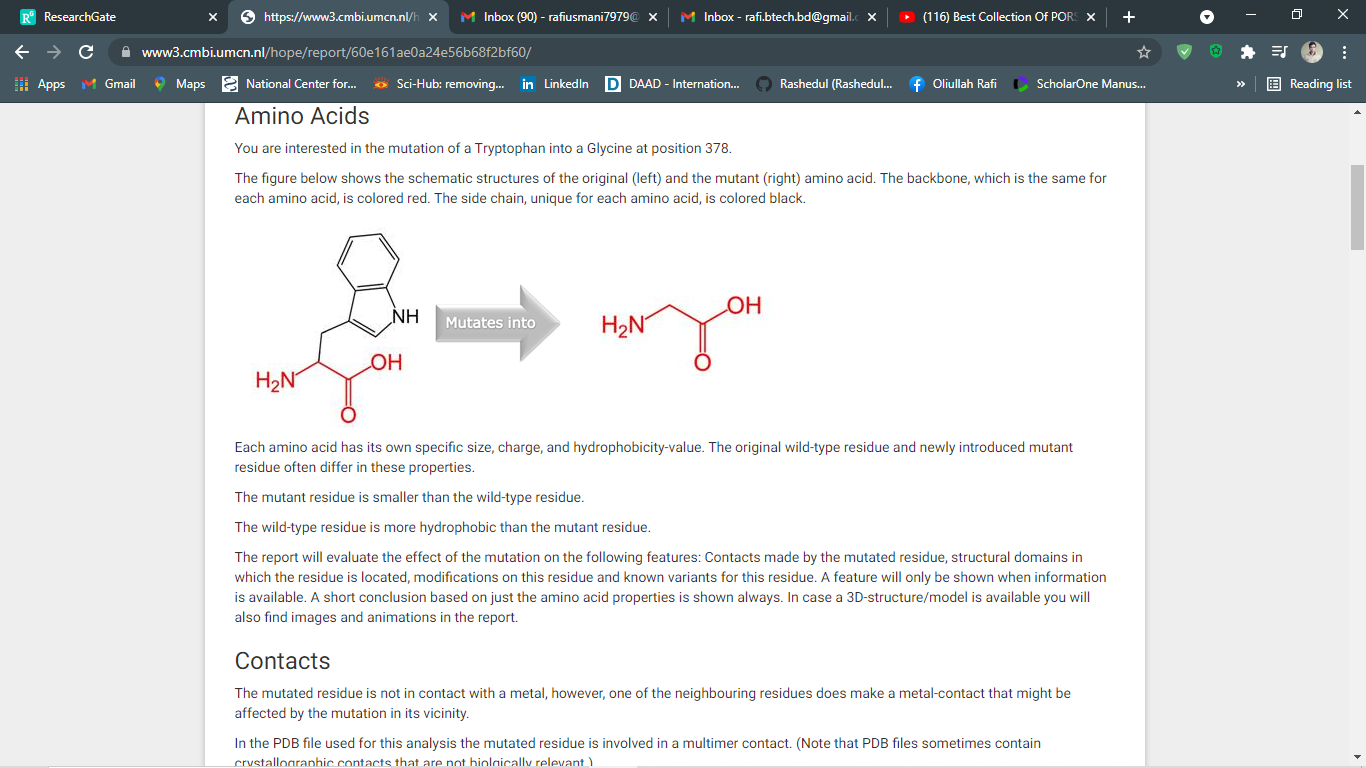 | -Each amino acid has its own specific size, charge, and hydrophobicity-value.  -The original wild-type residue and newly introduced mutant residue often differ in these properties.  -The mutant residue is smaller than the wild-type residue.  -The wild-type residue is more hydrophobic than the mutant residue. |
| **F379V** | 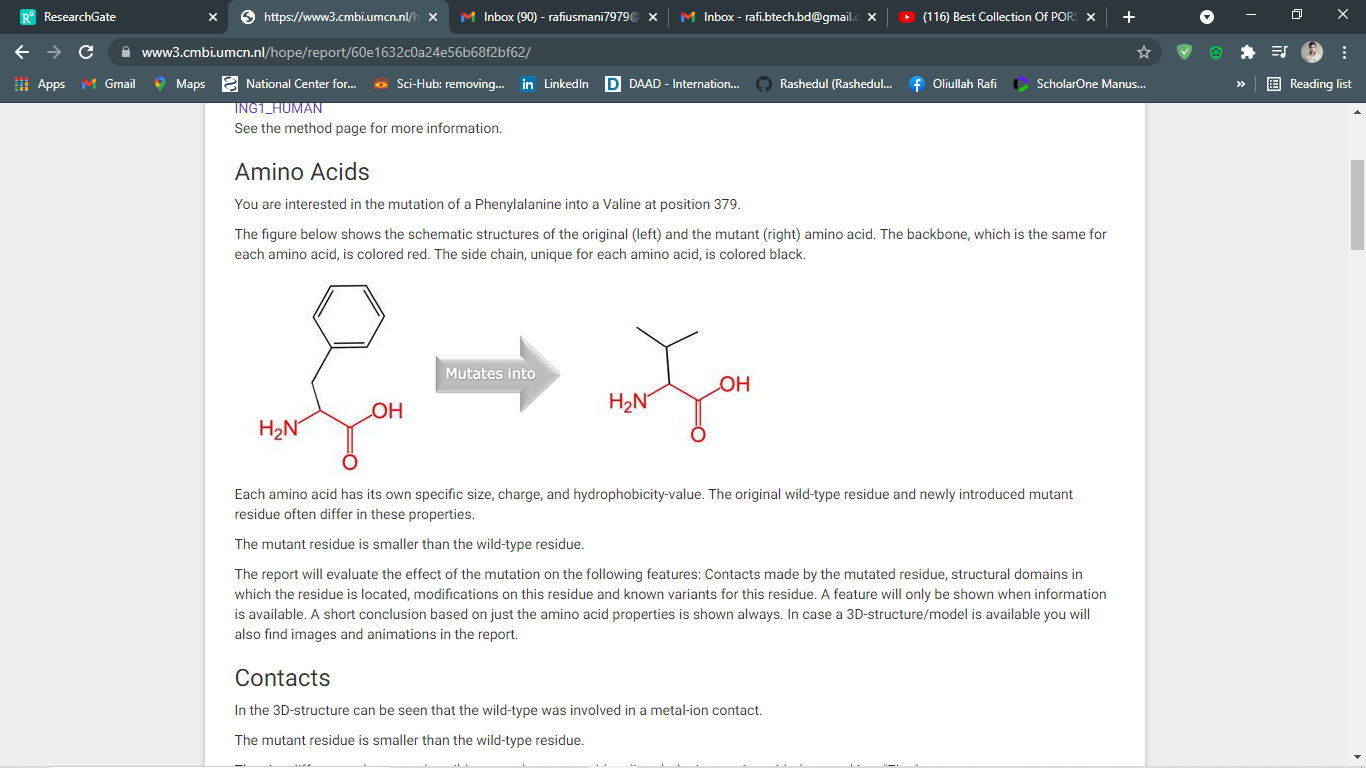 | -Each amino acid has its own specific size, charge, and hydrophobicity-value.  - The original wild-type residue and newly introduced mutant residue often differ in these properties.  -The mutant residue is smaller than the wild-type residue. |
| **S382L** | 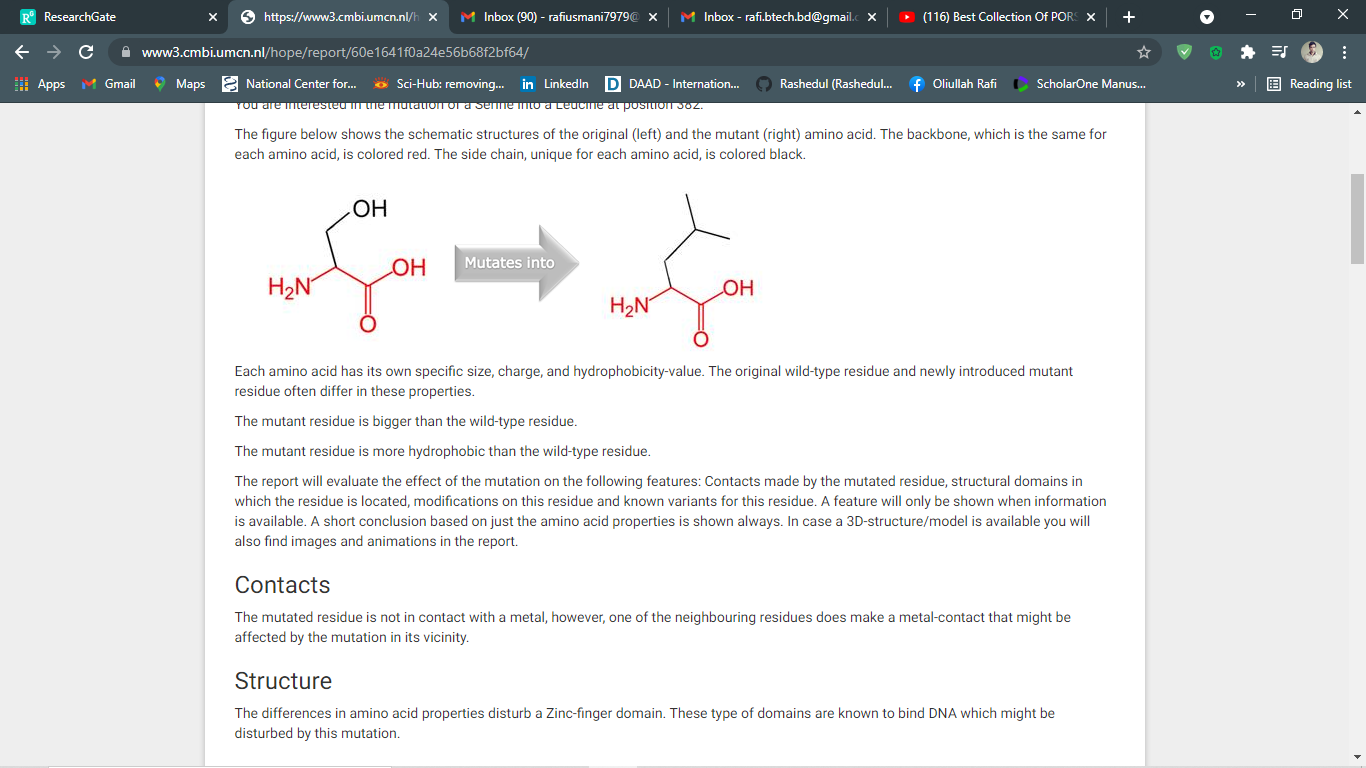 | -Each amino acid has its own specific size, charge, and hydrophobicity-value.  - The original wild-type residue and newly introduced mutant residue often differ in these properties.  -The mutant residue is bigger than the wild-type residue.  -The mutant residue is more hydrophobic than the wild-type residue. |
| **R400P** | 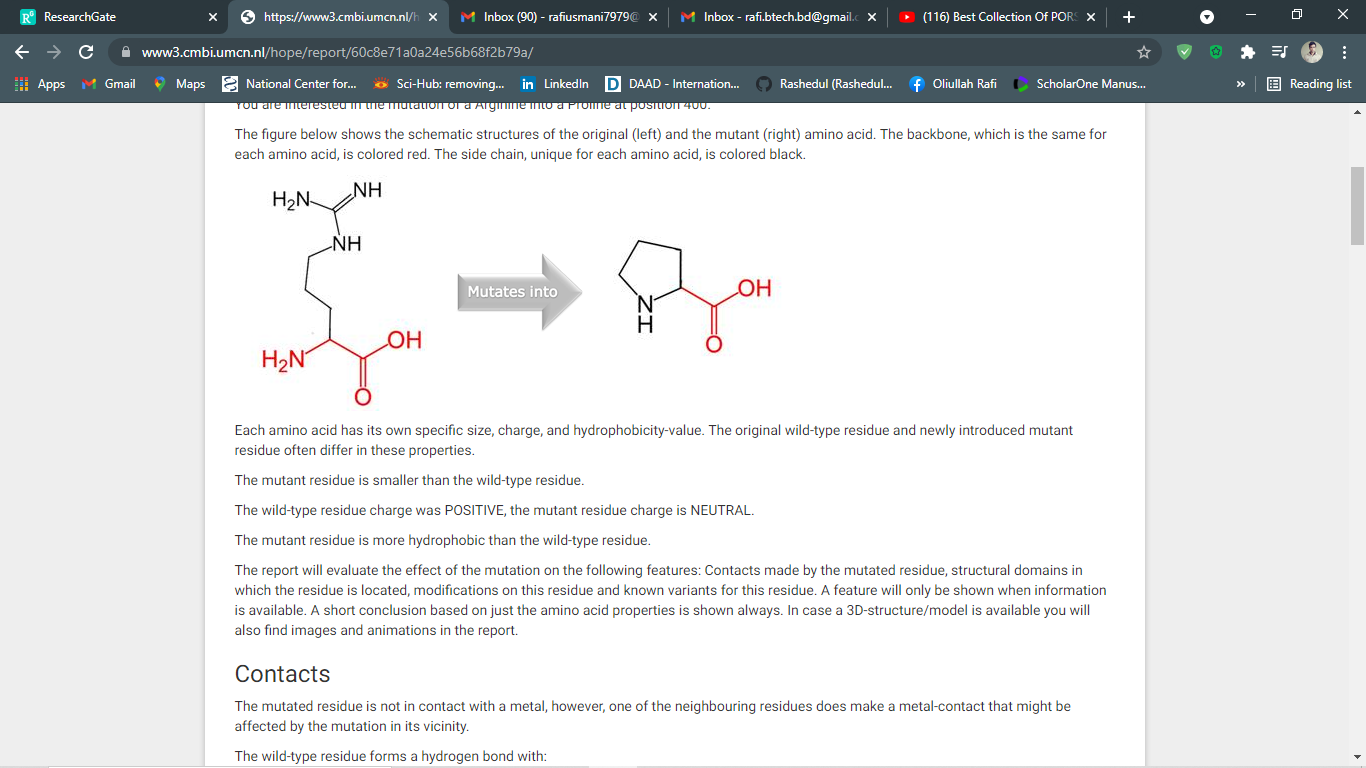 | -Each amino acid has its own specific size, charge, and hydrophobicity-value.  - The original wild-type residue and newly introduced mutant residue often differ in these properties.  -The mutant residue is smaller than the wild-type residue.  -The wild-type residue charge was POSITIVE, the mutant residue charge is NEUTRAL.  -The mutant residue is more hydrophobic than the wild-type residue. |

**Supplementary Table 2**: Secondary structural properties of wild-type and 6 mutant proteins. The secondary structure was evaluated by SOPMA and PSIPRED tools.

|  | **SOPMA** | | | | **PSIPRED** | | |
| --- | --- | --- | --- | --- | --- | --- | --- |
| **Variant** | Alpha helix | Extended strand | Beta turn | Random coil | Alpha helix | Beta strand | Coil |
| **Wild Type** | 31.75% | 11.61% | 4.27% | 52.37% | 33.88% | 6.63% | 59.47% |
| **C358S** | 32.23% | 11.61% | 4.50% | 51.66% | 33.64% | 6.63% | 59.71% |
| **C374G** | 31.04% | 11.85% | 4.74% | 52.37% | 30.56% | 6.635% | 62.79% |
| **R400P** | 34.83% | 11.85% | 4.98% | 48.34% | 32.93% | 6.87% | 60.18% |
| **S382L** | 30.81% | 11.61% | 4.74% | 52.84% | 34.59% | 6.39% | 59% |
| **F379V** | 30.33% | 12.09% | 4.74% | 52.84% | 35% | 6.63% | 58.29% |
| **W378G** | 31.28% | 11.85% | 4.74% | 52.13% | 35.78% | 5.21% | 59% |

**Supplementary Table 3:** Structural quality assessment of the refined model generated by the GalaxyRefine tool, including GDT-HA, RMSD, MolProbity , clash score, poor rotamers, and Ramachandran favored region.

| Model | GDT-HA | RMSD | MolProbity | Clash score | Poor rotamers | Rama favored |
| --- | --- | --- | --- | --- | --- | --- |
| Wild Type | 0.8460 | 0.731 | 0.992 | 1.9 | 0.0 | 97.9 |
| C358S | 0.8679 | 0.698 | 1.212 | 2.2 | 0.6 | 96.7 |
| C374G | 0.8637 | 0.716 | 1.106 | 2.8 | 0.3 | 97.9 |
| W378G | 0.8620 | 0.695 | 1.269 | 2.7 | 0.6 | 96.7 |
| F379V | 0.8549 | 0.737 | 1.238 | 2.1 | 0.9 | 96.2 |
| S382L | 0.8560 | 0.765 | 1.243 | 3.0 | 0.3 | 97.1 |
| R400P | 0.8750 | 0.658 | 0.971 | 1.5 | 0.6 | 97.6 |

**Supplementary Table 4**: RMSD value and TM-score of 6 most damaging nsSNPs of ING1 protein using TM-Align

| **AA change** | **Aligned length** | **RMSD** | **TM-score** |
| --- | --- | --- | --- |
| **C358S** Superposition to **wt** | 163 | 4.12 | 0.32110 |
| **C374G** Superposition to **wt** | 299 | 5.81 | 0.50531 |
| **W378G** Superposition to **wt** | 214 | 4.47 | 0.42262 |
| **F379V** Superposition to **wt** | 197 | 6.08 | 0.33046 |
| **S382L** Superposition to **wt** | 285 | 5.39 | 0.50962 |
| **R400P** Superposition to **wt** | 205 | 5.46 | 0.36935 |

| **predicts structural changes caused by missense variants in protein tertiary structures and binary complexes.** | | | |
| --- | --- | --- | --- |
| **Mutants** | **Residue type** | **Prediction** | **Effect** |
| Y355A | Exposed | Benign | No |
| **C358S** | Exposed | Benign | No |
| N359S | Exposed | Benign | No |
| V361I | Exposed | Benign | No |
| G364V | Exposed | Damaging | **Gly in a bend:** This substitution replaces glycine originally located in a bend curvature. |
| **C374G** | Buried | Benign | No |
| **W378G** | Exposed | Benign | No |
| W378A | Exposed | Benign | No |
| **F379V** | Buried | Damaging | This substitution triggers a **clash alert**. The local clash score for the wild-type structure is 16.75 and the local clash score for the variant structure is 54.22. |
| **S382L** | Exposed | Benign | No |
| **R400P** | Exposed | Damaging | **Secondary structure altered:** This substitution changes 'H' (4-turn helix) to 'T' (hydrogen-bonded turn). |

**Supplementary Table 5:** Assessment of 11 missense mutations on proteins tertiary structure.

**#Highlighted** are the SNPs we discovered in our analysis. Other SNPs are from previously discovered in literature.

**Supplementary Table 6:** Predicted Binding affinity changes upon mutation in the ING1 (PHD – H3K4me3) complex**.**

| **S. No** | **Mutation** | **ΔΔGBind (kcal/mol)** | **Solvent accessibility (in partner(s))** | **Solvent accessibility (in complex)** | **Interface** |
| --- | --- | --- | --- | --- | --- |
| 1 | Y 355 A | **1.42** | 33.36 % | 24.07 % | Yes |
| 2 | **C 358 S** | 0.05 | 27.38 % | 27.38 % | No |
| 3 | N 359 S | 0.15 | 65.26 % | 65.26 % | No |
| 4 | V 361 I | 0.06 | 46.81 % | 46.81 % | No |
| 5 | G 364 V | **0.96** | 84 % | 49.94 % | Yes |
| 6 | **C 374 G** | 0.71 | 9.58 % | 9.58 % | No |
| 7 | **W 378 G** | **3.17** | 36.8 % | 7.13 % | Yes |
| 8 | W 378 A | **2.55** | 36.8 % | 7.13 % | Yes |
| 9 | **F 379 V** | 0.48 | 1.8 % | 1.8 % | No |
| 10 | **S 382 L** | 0.12 | 69.34 % | 69.34 % | No |
| 11 | **R400P** | 0.50 | 46.88 % | 46.88 % | No |

**#Highlighted** are the SNPs we discovered in our analysis. Other SNPs are from previously discovered in literature.

| **Protein-Protein Affinity Change Upon Mutation** | | | |
| --- | --- | --- | --- |
| **Mutants** | **Residue relative solvent accessibility, RSA (%)** | **Protein-Protein Affinity Change (ΔΔG)** | **Outcome** |
| Y355A | 34.9 | -0.616 | Destabilizing |
| **C358S** | 34.2 | -1.172 | Destabilizing |
| N359S | 91.6 | 0.173 | Stabilizing |
| V361I | 23.0 | -0.579 | Destabilizing |
| G364V | 112.4 | 0.036 | Stabilizing |
| **C374G** | 0.6 | -0.746 | Destabilizing |
| **W378G** | 27.2 | -0.703 | Destabilizing |
| W378A | 27.2 | -0.793 | Destabilizing |
| **F379V** | 0.0 | -0.512 | Destabilizing |
| **S382L** | 81.8 | -0.034 | Destabilizing |
| **R400P** | 78.3 | -0.469 | Destabilizing |

**Supplementary Table 7:** Impact of mutation-induced changes on the Protein-protein binding affinity.

**#Highlighted** are the SNPs we discovered in our analysis. Other SNPs are from previously discovered in literature.

**Supplementary Table 8:** Prediction of Phosphorylation Sites by NetPhos 3.1 and GPS 3.0.

| **NetPhos 3.1** | | | | **GPS 3.0** | | | |
| --- | --- | --- | --- | --- | --- | --- | --- |
|  | **Position** | **Score** | **Kinase** | **Position** | **Score** | **Cutoff** | **Kinase** |
| **Threonine (T)** | 205 | 0.729 | PKC | 134 | 21.721 | 20.98 | AGC/DMPK/GEK |
|  | 318 | 0.990 | unsp | 178 | 2.923 | 2.819 | AGC/DMPK/GEK/DMPK |
|  | 326 | 0.938 | PKC |  |  |  |  |
|  | 406 | 0.525 | CKII |  |  |  |  |
| **Serine (S)** | 2* | 0.517 | CKII | 2* | 4.511 | 2.819 | AGC/DMPK/GEK/DMPK |
|  | 10* | 0.941 | unsp | 10* | 4.755 | 2.819 | AGC/DMPK/GEK/DMPK |
|  | 40* | 0.638 | PKC | 69* | 3.563 | 2.819 | AGC/DMPK/GEK/DMPK |
|  | 42 | 0.632 | unsp | 414* | 2.967 | 2.819 | AGC/DMPK/GEK/DMPK |
|  | 45 | 0.974 | unsp | 25 | 23.583 | 20.98 | AGC/DMPK/GEK |
|  | 59 | 0.600 | PKA | 175* | 23.904 | 20.98 | AGC/DMPK/GEK |
|  | 68 | 0.768 | PKC | 290* | 24.47 | 20.98 | AGC/DMPK/GEK |
|  | 69* | 0.525 | cdc2 | 109* | 35.982 | 34.253 | AGC/DMPK/ROCK/ROCK1 |
|  | 78 | 0.553 | cdc2 | 333* | 44.992 | 34.253 | AGC/DMPK/ROCK/ROCK1 |
|  | 87 | 0.539 | cdc2 | 342* | 42.369 | 34.253 | AGC/DMPK/ROCK/ROCK1 |
|  | 92 | 0.705 | unsp | 156* | 25.098 | 23.835 | AGC/DMPK/ROCK/ROCK2 |
|  | 94 | 0.629 | PKG | 293 | 11.688 | 11.636 | AGC/DMPK/ROCK |
|  | 102 | 0.603 | unsp |  |  |  |  |
|  | 109* | 0.882 | unsp |  |  |  |  |
|  | 123 | 0.992 | unsp |  |  |  |  |
|  | 128 | 0.991 | unsp |  |  |  |  |
|  | 131 | 0.570 | p38MAPK |  |  |  |  |
|  | 157 | 0.928 | unsp |  |  |  |  |
|  | 156* | 0.544 | unsp |  |  |  |  |
|  | 175* | 0.540 | cdc2 |  |  |  |  |
|  | 202 | 0.987 | unsp |  |  |  |  |
|  | 224 | 0.895 | unsp |  |  |  |  |
|  | 236 | 0.605 | DNAPK |  |  |  |  |
|  | 251 | 0.991 | unsp |  |  |  |  |
|  | 269 | 0.976 | unsp |  |  |  |  |
|  | 290* | 0.988 | unsp |  |  |  |  |
|  | 307 | 0.996 | unsp |  |  |  |  |
|  | 327 | 0.995 | unsp |  |  |  |  |
|  | 333* | 0.987 | unsp |  |  |  |  |
|  | 342* | 0.997 | unsp |  |  |  |  |
|  | 414* | 0.994 | unsp |  |  |  |  |
| **Tyrosine (Y)** | 8 | 0.905 | unsp |  |  |  |  |
|  | 198 | 0.660 | unsp |  |  |  |  |
|  | 355 | 0.901 | unsp |  |  |  |  |

***Common in both NetPhos 3.1 and GPS 3.0.**

**Supplementary Table 9:** Prediction of Ubiquitylation site by BDM-PUB server.

|  |  | BDM-PUB |  |
| --- | --- | --- | --- |
| Peptide | Position | Score | Threshold |
| WGRAWPWKQILKELD | 188 | 1.58 | 0.3 |
| RETDGAQKRRMLHCV | 210 | 1.41 | 0.3 |
| DTAGNSGKAGADRPK | 271 | 2.14 | 0.3 |
| KAGADRPKGEAAAQA | 278 | 1.18 | 0.3 |
| EAAAQADKPNSKRSR | 287 | 3.21 | 0.3 |
| QADKPNSKRSRRQRN | 291 | 2.76 | 0.3 |
| DGASGTPKEKKAKTS | 320 | 2.51 | 0.3 |
| ASGTPKEKKAKTSKK | 322 | 3.15 | 0.3 |
| SGTPKEKKAKTSKKK | 323 | 3.91 | 0.3 |
| TPKEKKAKTSKKKKR | 325 | 4.08 | 0.3 |
| EKKAKTSKKKKRSKA | 328 | 3.55 | 0.3 |
| KKAKTSKKKKRSKAK | 329 | 4.20 | 0.3 |
| KAKTSKKKKRSKAKA | 330 | 3.20 | 0.3 |
| AKTSKKKKRSKAKAE | 331 | 5.30 | 0.3 |
| SKKKKRSKAKAEREA | 334 | 4.16 | 0.3 |
| KKKRSKAKAEREASP | 336 | 2.45 | 0.3 |
| SCVGLNHKPKGKWYC | 389 | 0.60 | 0.3 |
| LNHKPKGKWYCPKCR | 393 | 0.76 | 0.3 |
| KGKWYCPKCRGENEK | 398 | 0.64 | 0.3 |
| KCRGENEKTMDKALE | 405 | 1.17 | 0.3 |
| ENEKTMDKALEKSKK | 409 | 1.45 | 0.3 |
| TMDKALEKSKKERAY | 413 | 2.01 | 0.3 |
| DKALEKSKKERAYNR | 415 | 2.23 | 0.3 |
| KALEKSKKERAYNR | 416 | 1.25 | 0.3 |

**Supplementary Table 10.xlsx :** Prediction of glycosylation sites by NetOGlyc 4.0 server

**Supplementary Table 11:** Functional protein partners of ING1 predicted by STRING

| **S.NO** | **Functional Partners** | **Coexpression** | **Experiments** | **Text mining** | **Score** |
| --- | --- | --- | --- | --- | --- |
| 1 | HDAC1 | Yes | Yes | Yes | 0.998 |
| 2 | SIN3A | Yes | Yes | Yes | 0.997 |
| 3 | TP53 | No | Yes | Yes | 0.992 |
| 4 | SP30 | No | Yes | Yes | 0.991 |
| 5 | HDAC2 | Yes | Yes | Yes | 0.961 |
| 6 | HIS2H3PS2T | Yes | Yes | Yes | 0.95 |
| 7 | HIST2H3D | Yes | Yes | Yes | 0.948 |
| 8 | GADD45A | NO | No | Yes | 0.904 |
| 9 | RBBP7 | Yes | Yes | Yes | 0.89 |
| 10 | KIAA0101 | No | Yes | Yes | 0.892 |

**Supplementary Table 12:** Interaction of ING1 with other genes and their network group.

| **Gene 1** | **Gene 2** | **Weight** | **Network group** |
| --- | --- | --- | --- |
| SIN3A | ING1 | 0.0029123677 | Co-expression |
| TM9SF2 | ING1 | 0.024836443 | Co-expression |
| TM9SF2 | ING1 | 0.019348703 | Co-expression |
| TM9SF2 | ING1 | 0.019478874 | Co-expression |
| SAP30 | ING1 | 0.014452301 | Co-expression |
| SINHCAF | ING1 | 0.1538068 | Physical Interactions |
| HDAC1 | ING1 | 0.0335607 | Physical Interactions |
| SAP30 | ING1 | 0.09751421 | Physical Interactions |
| SIN3A | ING1 | 0.15269588 | Physical Interactions |
| PCNA | ING1 | 0.3218301 | Physical Interactions |
| BRMS1 | ING1 | 0.03019961 | Physical Interactions |
| PCNA | ING1 | 0.00635196 | Physical Interactions |
| NQO1 | ING1 | 0.042504556 | Physical Interactions |
| HDAC1 | ING1 | 0.003938534 | Physical Interactions |
| SAP30 | ING1 | 0.025344681 | Physical Interactions |
| SIN3A | ING1 | 0.007992193 | Physical Interactions |
| SINHCAF | ING1 | 0.17516112 | SINHCAF |
| HDAC1 | ING1 | 0.11482719 | Physical Interactions |
| NQO1 | ING1 | 0.56837595 | Physical Interactions |
| PCNA | ING1 | 0.2256411 | Physical Interactions |
| HDAC1 | ING1 | 0.3811788 | Physical Interactions |
| PCLAF | ING1 | 0.3574162 | Physical Interactions |
| CTRL | ING1 | 0.94047856 | Physical Interactions |
| PCNA | ING1 | 0.16277613 | Physical Interactions |
| HDAC1 | ING1 | 0.100847326 | Physical Interactions |
| SAP30 | ING1 | 0.20450455 | Physical Interactions |
| CTRL | ING1 | 0.5753348 | Physical Interactions |
| PCNA | ING1 | 0.03013113 | Physical Interactions |
| NQO1 | ING1 | 0.30153027 | Physical Interactions |
| ING5 | ING1 | 0.018610263 | Shared protein domains |
| ING2 | ING1 | 0.018452723 | Shared protein domains |
| ING4 | ING1 | 0.018566217 | Shared protein domains |
| ING3 | ING1 | 0.018627247 | Shared protein domains |
| ING5 | ING1 | 0.25 | Shared protein domains |
| ING2 | ING1 | 0.25 | Shared protein domains |
| ING4 | ING1 | 0.25 | Shared protein domains |
| ING3 | ING1 | 0.25 | Shared protein domains |
| BRMS1 | ING1 | 0.70222116 | Predicted |
| TOLLIP | ING1 | 0.36941838 | Predicted |
| GTF2A1 | ING1 | 0.40508935 | Predicted |
| RXFP1 | ING1 | 0.3686781 | Predicted |
| PYCR3 | ING1 | 0.3686781 | Predicted |
| PPME1 | ING1 | 0.3686781 | Predicted |
| PGRMC1 | ING1 | 0.3686781 | Predicted |

**Supplementary Materials**

****Supplementary Material 1:** Prediction result of 347 ING1 nsSNPs by 12 computational tools (PolyPhen-2, Pmut, Mutation Assessor, fathmm, Condel, PROVEAN, SNPs & GO, Panther-PSEP, PhD-SNP, SIFT, SNAP, Meta-SNP).
